# Authors’ Response to Peer Review of “Associations Between IT Job Stressors and Anxiety, Depression, and Stress: Cross-Sectional Study”

**DOI:** 10.2196/91437

**Published:** 2026-03-03

**Authors:** Edlin Garcia Colato, Nianjun Liu, Angela Chow, Catherine M Sherwood-Laughlin, Jonathan T Macy

**Affiliations:** 1 Department of Health and Wellness Design, School of Public Health, Indiana University Bloomington, 1025 E 7th St, Bloomington, IN, 47405, United States, 1 812-855-3102; 2 Department of Epidemiology and Biostatistics, School of Public Health, Indiana University Bloomington, Bloomington, IN, United States; 3 Department of Applied Health Science, School of Public Health, Indiana University Bloomington, Bloomington, IN, United States

**Keywords:** survey, association, occupational health, mental health, stressors, IT, IT professionals, United States, workplace, depression, anxiety, stress, help-seeking, health literacy

*This is the authors’ response to the peer review of “Associations Between IT Job Stressors and Anxiety, Depression, and Stress: Cross-Sectional Study.” [1]*

## Round 1 Review

### Reviewer AD [2]

#### General Comments

*Although the data basis of this study is not very reliable due to some methodological limitations (cross-sectional design, online survey, self-reporting only, self-selection bias), the study does provide some interesting insights. I have the following comments to make.*

#### Specific Comments

##### Major Comments

*What I miss most is a more differentiated discussion of the assumed mediation by mental health literacy (MHL). In my view, it is not necessarily plausible. It would be just as plausible to assume that people with high MHL are more competent in knowing what to do by themselves. In addition, MHL itself can also have a protective function, in that people with high MHL also know what they can do by themselves to protect themselves in the event of a high workload and apply any compensatory measures (eg, relaxation).*

**Response:** Thank you for your thoughtful feedback regarding the assumed mediation by MHL. We appreciate your point that the plausibility of MHL as a mediator warrants a more nuanced discussion. In the revised manuscript, we have expanded this section to acknowledge the limitations in interpretation.

##### Minor comments

*1. Some information on the analysis should be added to the abstract.*

**Response:** We have updated it to include the following: “Descriptive statistics, regression models, and mediation analyses were conducted for CESD-10, GAD-7, and PSS-10.”

*2. “Anxiety” should also be included in the keywords.*

**Response:** Thank you for the suggestion, we have included “anxiety” as a keyword.

*3. With regard to the excluded cases, no cases are mentioned that were excluded due to conspicuous response behavior (eg, monotonous patterns) or too rapid completion (“speeders”). Why?*

**Response:** We are aware that excluding cases for monotonous patterns or too-rapid completion is a common practice to ensure data validity. We have updated our text to explain that rapidly completed surveys were marked as invalid and were thus removed from the final sample. However, in our review of our data, there was no evidence of monotonous completion. All retained observations had completion times that were within the expected ranges, and the response patterns showed sufficient variability.

We added the following text: “Review of the data showed no evidence of conspicuous response behavior. Outliers in average completion time of the survey that showed the survey was completed in only a few minutes however were excluded. A total of 388 (84.3%) of the remaining 460, who provided consent and were determined to be valid responses, completed the survey.”

*4. Please add information on how many people in total were initially contacted.*

**Response:** We have added that approximately 2336 individuals were contacted.

*5. A core element of the study is the initial identification of IT-specific stressors. Here, further information on the criteria for the selection of the experts and the methodological procedure for capturing the stressors is essential (interviews? focus group? workshop?).*

**Response:** We added the following text: “IT experts were selected based on predefined criteria, including their professional qualifications and practical experience. Interviews were conducted with the IT experts, allowing for the development of a comprehensive list of stressors.”

*6. The study just assessed the intention to seek help. Did it also assess whether professional help had been sought in the past 12 months? If not, why not? This would have been very easy to capture and a much more reliable criterion than just intentions.*

**Response:** Our study focused on assessing intention to seek professional help as a proxy for help-seeking behavior, which aligns with the theoretical framework underpinning our research. We did not include a measure of actual help-seeking within the past 12 months. The main reason was that the objective was to examine prospective behavioral tendencies rather than retrospective behaviors. While we acknowledge that past behavior is an important factor, our design prioritized capturing motivational aspects to inform future help-seeking decisions.

*7. Please add a table with the most important information about the sample.*

**Response:** We added back the sociodemographic table, Table 1 (“Characteristics by Sex”), in the Results section.

*8. Please also add a table with the frequencies of the individual stressors as well as the distribution of multiple stressors (ie, how often people reported 1 stressor, 2 stressors, etc, up to 12 stressors). This is also interesting, as the effect of multiple stressors appears to be surprisingly small. The type of stressor therefore seems to be more decisive than the frequency/diversity.*

**Response:** Thank you for this suggestion, we have created the requested table and have included it in the appendices as supplemental material.

*9. Table 1 has a different font type, please adjust.*

**Response:** Thanks for pointing this out. We removed the original Table 1 and instead provided the odds ratios, CIs, and *P* values in the text, and we have reviewed the other tables to ensure they use consistent font types.

*10. Some of the terms in Table 2 are written inconsistently (eg, “p-value”/“P value”).*

**Response:** We have updated all mentions of *P* values throughout the tables and text to ensure consistency.

*11. Please add a legend beneath Table 2, explaining the abbreviations of the measures.*

**Response:** Thank you, we have updated the table to include full definitions of abbreviations of the measures, as suggested.

*12. The discussion basically only addresses why mediation shows no effect with regard to stress, but not why this is also the case with anxiety. Please also address this.*

**Response:** Thank you for bringing this to our attention. We revised the discussion to include possible reasons why mediation was not observed for anxiety. We added the following text: “The lack of mediation found between MHL and anxiety and stress could be due to the measurement timing or the cross-sectional design that limited the ability to detect indirect effects for both anxiety and stress. Future research using longitudinal designs and alternative mediators could clarify whether these null findings reflect a true absence of mediation or methodological constraints.”

*13. Incidentally, the mediation effect for depression should not be overestimated. The effect just tips significance and the size of the indirect effect is rather small relative to the huge direct effect.*

**Response:** Thank you for highlighting this point. We agree that the mediation effect for depression should not be overestimated. The mediation suggests a pathway worth noting. We updated the text to clarify that the indirect effect is modest, as follows: “The mediation results suggests a pathway worth noting between MHL and help-seeking for depression; although the indirect effect is modest. MHL had only a partial mediation for depression, but not for anxiety or stress.”

*14. The assessment of MHL by self-report is not necessarily a limitation per se, as there are both objective and subjective concepts in MHL. The question is rather which version is the more suitable for operationalization for testing your hypotheses.*

**Response:** Thank you for pointing that out. MHL can be conceptualized both objectively and subjectively. We chose a self-report measure because our hypotheses focused on individuals’ perceived ability to recognize and respond to mental health issues, which aligns with the subjective dimension of MHL. However, we agree that discussing why this operationalization was most suitable would strengthen the manuscript. We specifically stated that “MHL-W is the only validated MHL instrument that is specific to the workplace making it the most suitable for this type of research.”

*15. The references are still very inconsistent, eg, some journal titles are abbreviated/others are not; some references lack information (eg, Northwave—Where did “In” appear?) or the year of publication (eg, for Boehm et al [3]). Please check the reference list manually throughout.*

**Response:** All references have been updated with the *JMIRx Med* Endnote tool and manually reviewed for consistency and accurate information.

## Abbreviations

MHL: mental health literacy
